# Single‐Dose Pharmacokinetics and Safety of Atogepant in Adults With Hepatic Impairment: Results From an Open‐Label, Phase 1 Trial

**DOI:** 10.1002/cpdd.916

**Published:** 2021-01-27

**Authors:** Ramesh Boinpally, Abhijeet Jakate, Matthew Butler, Lisa Borbridge, Antonia Periclou

**Affiliations:** ^1^ AbbVie Madison New Jersey USA

**Keywords:** atogepant, calcitonin gene‐related peptide receptor antagonists, hepatic impairment, migraine disease, pharmacokinetics

## Abstract

Atogepant is a selective oral calcitonin gene‐related peptide receptor antagonist in development for migraine prevention. Here, we report the pharmacokinetics (PK) and safety of single‐dose 60 mg atogepant in participants with severe, moderate, or mild hepatic impairment and matched participants with normal hepatic function from an open‐label, parallel‐group, single‐dose phase 1 trial. Thirty‐two participants aged 45 to 72 years were enrolled, which included 8 each with severe, moderate, mild, or no hepatic impairment. All participants completed the study. Atogepant was rapidly absorbed (median time to maximum plasma concentration, ∼2 hours) with an apparent terminal elimination half‐life of ∼11 hours. Compared with participants with normal hepatic function, the change in maximum plasma concentrations of atogepant were –4%, –12%, and +9% in participants with severe, moderate, or mild hepatic impairment, respectively. Overall systemic exposures to atogepant were 15% to 38% higher in participants with hepatic impairment compared with those with normal hepatic function, but these differences are not expected to be clinically relevant given the established safety profile of atogepant. Only 1 adverse event was reported: mild rhinorrhea in a participant with moderate hepatic impairment. Overall, atogepant was safe and not associated with any clinically relevant change in PK in participants with severe, moderate, or mild hepatic impairment.

Migraine is a complex chronic disease with recurrent attacks that imposes substantial burden on individuals.^1^ Because of its high global prevalence, migraine is the leading cause of years lived with disability in those younger than 50 years of age.^2^ Current oral treatment options for the prevention of migraine attacks often fail to meet treatment goals or are poorly tolerated.^3^, ^4^ As a result, a substantial number of people discontinue preventive treatment without adequately reducing migraine frequency or severity.^3^ New preventive treatments with a more favorable efficacy and tolerability profile are needed to reduce migraine frequency and ease the substantial burden and disability it imposes on individuals and society.

The calcitonin gene‐related peptide (CGRP) is an inflammatory mediator and a potent vasodilator that triggers migraine attacks.^5^, ^6^ Targeted treatments, specifically antagonists to block CGRP or its receptor, have been developed for the prevention or treatment of migraine attacks.^5^ Small‐molecule CGRP receptor antagonists (gepants), such as ubrogepant and rimegepant, have been recently approved for the acute treatment of migraine attacks, and monoclonal antibodies blocking the CGRP pathway have been approved for the prevention of migraine.^6^, ^7^ For people who fail to meet treatment goals with existing oral preventive treatments and those who prefer an oral route of administration, small‐molecule CGRP receptor antagonists may be a valuable preventive treatment option.

Telcagepant was the first gepant to be developed for use in migraine, but was associated with elevated levels of alanine aminotransferase (ALT) with daily dosing, and its development was consequently discontinued.^8^, ^9^ MK‐3207 was similarly associated with elevated ALT levels and delayed liver‐test abnormalities, which caused discontinuation of its clinical development.^10^ The clinical development programs for several first‐generation gepants tested in clinical trials were discontinued because of issues with drug‐induced liver injury (DILI),^11^ although all had met their primary efficacy end points.^10^, ^12^, ^13^

Atogepant is a potent, selective, competitive, second‐generation oral CGRP receptor antagonist that is in development for preventive treatment of migraine (ClinicalTrials.gov NCT03700320, NCT03777059, NCT03855137, NCT03939312). The half‐life of atogepant is approximately 11 hours, with a T_max_ of 1 to 2 hours and no evidence of significant accumulation with repeated daily dosing. It was designed to minimize the potential for reactive metabolites to reduce the risk of DILI seen with other members of this drug class. Atogepant is eliminated primarily via hepatic metabolism and biliary secretion. Hepatic metabolism occurs predominantly through oxidation via cytochrome P450 (CYP) 3A4 and to some extent via CYP2D6. Further in vitro studies showed that atogepant is not a potent reversible or time‐dependent inhibitor of CYP3A4 (IC_50_ > 100 μM) or UDP‐glucuronosyltransferase 1A1. In addition, atogepant does not induce CYP1A2 or CYP2B6 in vitro and does not inhibit P‐glycoprotein. In a 12‐week randomized, placebo‐controlled, multidose phase 2/3 clinical trial, atogepant was safe, with demonstrated efficacy in migraine prevention at all doses tested compared with placebo.^14^, ^15^ No evidence of a deleterious effect of atogepant on liver safety has been observed in preclinical or clinical studies to date, including a 28‐day hepatic safety study^16^ and a phase 1 study assessing drug‐drug interactions (DDIs) with oral contraceptives (OCs).^17^

The objective of this study was to assess the pharmacokinetics (PK), safety, and tolerability profiles of single‐dose oral atogepant in participants with normal and impaired hepatic function.

## Methods

### Study Design

This was a multicenter, nonrandomized, open‐label, parallel‐group, single‐dose phase 1 trial. Adult participants were screened for eligibility, and those with hepatic impairment were categorized based on Child‐Pugh score^18^ within 14 days before atogepant administration. Participants were not screened for a history of migraine. Participants were admitted to the study center for days – 1 through 4, had an end‐of‐study visit within 7 days of the final PK sample collection, and had a follow‐up visit on day 30 (±2 days). Under fasted conditions, participants received a single oral 60‐mg tablet of atogepant on day 1. Blood samples for PK testing were collected at prespecified times from day 1 through day 4, and safety was monitored throughout the study to the follow‐up visit on day 30.

This trial was conducted in accordance with the Declaration of Helsinki, in compliance with the guidance from the International Council on Harmonisation and Good Clinical Practice. The study was conducted at Clinical Pharmacology of Miami, Inc. (Miami, Florida), and Orlando Clinical Research Center (Orlando, Florida), and the study protocol was approved by the Internal Review Board at IntegReview IRB (Austin, Texas). Participants provided informed consent prior to the initiation of any trial‐specific procedures.

### Participants

Participants with and without hepatic impairment were recruited. Inclusion criteria for all participants were aged 18 to 80 years (inclusive), nonsmoker or light smoker (fewer than 10 cigarettes per day within 1 week before atogepant administration), body mass index (BMI) of at least 18 kg/m^2^ and no higher than 42 kg/m^2^, and a sitting pulse rate of at least 50 bpm and no higher than 100 bpm during screening. Key exclusion criteria included known hypersensitivity to any CGRP receptor antagonist; an abnormal electrocardiogram (ECG) result; exposure to hepatitis B virus, hepatitis C virus, or HIV; and abnormal and clinically significant results from any screening tests (eg, physical, laboratory, medical history). Participants with hepatic impairment had chronic liver disease and/or cirrhosis documented by at least 1 of the following: liver biopsy with histologic findings consistent with cirrhosis; computerized tomographic or ultrasonographic evidence of liver disease with or without portal hypertension, physical examination and clinical and laboratory evidence of chronic liver disease, and colloid shift on a liver‐spleen scan. Participants with hepatic impairment could not have gastrointestinal hemorrhage because of esophageal varices, peptic ulcers, or Mallory‐Weiss syndrome within 6 months before day 1; an acute exacerbation of liver disease within 4 weeks of dosing; ascites requiring paracentesis within 1 week of dosing or during the study period; or a Child‐Pugh score greater than 13.

### Cohort Assignment and Initiation

Participants with hepatic impairment were categorized as having mild (Child‐Pugh score, 5 to 6), moderate (7 to 9), or severe (10 to 13) hepatic impairment at screening.^18^ Participants with mild hepatic impairment were enrolled first, and participants with moderate hepatic impairment were not enrolled until 4 participants with mild impairment had completed the trial. Then, participants with severe hepatic impairment were enrolled after 4 participants with moderate hepatic impairment had completed the trial. Participants with normal hepatic function were recruited after enrollment of the hepatically impaired groups to closely match participants in the groups with hepatic impairment in terms of age, sex, and weight.

### Outcomes

#### PK Assessments

The primary outcome was the assessment of PK parameters of atogepant derived from concentrations in plasma. Blood samples for PK evaluation were collected on day 1 before dosing and 0.5, 1, 1.5, 2, 3, 4, 6, 8, 12, and 16 hours after dosing and on day 2 (24 and 36 hours after dosing on day 1), day 3 (48 hours after dosing), and day 4 (72 hours after dosing). Levels of plasma protein‐bound atogepant were determined from samples collected on day 1 before dosing and 2 hours after dosing. Atogepant concentrations in plasma samples and plasma protein‐binding samples (following equilibrium dialysis) were analyzed by Keystone Bioanalytical, Inc. (North Wales, Pennsylvania) using validated liquid chromatography with tandem mass spectrometry methods similar to those described in a previous study.^17^

PK parameters assessed included maximum plasma atogepant concentration (C_max_), time to maximum plasma concentration (T_max_), area under the plasma concentration‐time curve from time 0 to time t (AUC_0‐t_) and from time 0 to infinity (AUC_0‐∞_), apparent terminal elimination half‐life (t_1/2_), and apparent total body clearance of drug from plasma after extravascular administration (CL/F).

#### Safety

Safety assessments included reports of adverse events (AEs) and serious AEs (SAEs); clinical laboratory assessments (hematology, coagulation, chemistry, urinalysis; samples collected at screening, on day –1, and at the end‐of‐study visits and chemistry assessment at the 30‐day follow‐up visit); and physical examinations, vital signs, and ECGs (evaluated at prespecified times through the study and at the end‐of‐study visit). AEs and SAEs were reported by participants or observed by study personnel at any time during the trial and through 30 days after atogepant administration. A laboratory alert for potential Hy's law cases was in place to monitor hepatic function from enrollment through 30 days after atogepant administration. Hy's law criteria included ALT or aspartate aminotransferase (AST) 3 or more times the upper limit of normal (ULN), total bilirubin 2 or more times the ULN, and alkaline phosphatase less than 2 times the ULN.

### Analytical Methods

Atogepant was extracted from human plasma via liquid‐liquid extraction. The analyte and internal standard (^2^H_3_‐atogepant) were analyzed by reverse‐phase chromatography (Zorbax Eclipse C18, 50 × 3 [Agilent, Santa Clara, California]; gradient mobile phase using 0.1% formic acid in water and acetonitrile) and positive ionization mode tandem mass spectrometry. The limit of quantitation for this method was 1 ng/mL using 50 μL of plasma, with a calibration range of 1 to 1000 ng/mL.

Plasma protein binding was determined using rapid equilibrium dialysis following manufacturer's instructions (Thermo Fisher Scientific, Waltham, Massachusetts). Analysis of samples was conducted using the method described above.

### Statistical Analysis

Descriptive statistics were calculated for continuous variables, and frequency distributions were presented for categorical variables. An interim analysis of PK and safety/tolerability data was conducted after the first 4 participants in the mild and moderate hepatic impairment groups had completed the study to inform further enrollment. Geometric means were calculated for C_max_, AUC_0‐t_, and AUC_0‐∞_, which were compared between each group with hepatic impairment and the group with normal hepatic function using a linear mixed‐effects model, with hepatic function group as a fixed effect. No imputations were made for missing data. Statistical analyses of safety outcomes were performed using SAS version 9.3 (SAS Institute, Cary, North Carolina), and PK analyses were conducted using Phoenix WinNonlin version 6.2 (Certara, L.P., Princeton, New Jersey).

## Results

### Participants

This trial was conducted at 2 study sites in the United States between November 1, 2016, and November 9, 2018. A total of 32 participants were enrolled in the trial (including 8 each with severe, moderate, or mild hepatic impairment and 8 participants with normal hepatic function), and all participants completed the study and were included in the safety population. Mean age was 58.8 years (range, 45‐72 years), most were male (68.8%) and white (87.5%), and mean BMI was 30.72 kg/m^2^ (Table 1). Demographic characteristics were generally similar across the 4 hepatic function groups.

**Table 1:** Demographics and Clinical Characteristics

Characteristic	Severe Hepatic Impairment (n = 8)	Moderate Hepatic Impairment (n = 8)	Mild Hepatic Impairment (n = 8)	Normal Hepatic Function (n = 8)
Age, mean y (SD) [range]	58.3 (8.0) [46–67]	60.4 (8.1) [45–72]	58.1 (4.6) [51–66]	58.4 (3.2) [55–63]
Sex (male), n (%)	5 (63)	5 (63)	7 (88)	5 (63)
Race, n (%)				
White	8 (100)	6 (75)	7 (88)	7 (88)
Black/African American	0	2 (25)	1 (13)	1 (13)
Ethnicity (Hispanic or Latino), n (%)	6 (75)	2 (25)	4 (50)	5 (63)
BMI (kg/m^2^), mean (SD) [range]	32.7 (5.0) [25.4–39.3]	30.6 (2.4) [26.5–34.5]	30.8 (7.0) [19.7–41.1]	28.8 (2.8) [24.9–32.8]

BMI, body mass index; SD, standard deviation.

### Atogepant Pharmacokinetics

**Figure 1:**
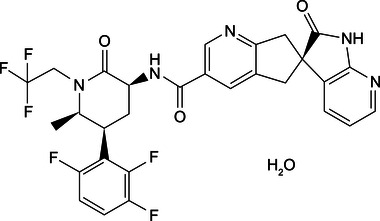
Chemical structure of atogepant.

**Figure 2:**
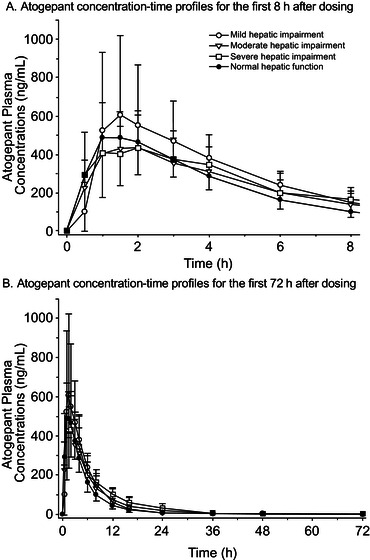
Plasma atogepant mean concentration‐time profiles following a single 60‐mg dose. Atogepant concentrations are shown on a linear scale for (A) the first 8 hours after administration and (B) the entire study (72 hours after administration). Mean values are shown for participants with severe (open squares), moderate (open triangles), and mild (open circles) hepatic impairment and for participants with normal hepatic function (closed circles). Error bars represent standard deviations. SD, standard deviation.

The chemical structure of atogepant is presented in Figure 1. Atogepant plasma concentration‐time profiles in participants with normal hepatic function and those with mild, moderate, or severe hepatic impairment are shown in Figure 2. Median T_max_ was similar among the 4 hepatic function groups (Table 2). Mean t_1/2_ of atogepant was marginally shorter in the severe hepatic impairment group compared with the other groups. Compared with participants with normal hepatic function, C_max_ was 9% higher in participants with mild hepatic impairment, and AUC_0‐∞_ was 38% higher in participants with severe hepatic impairment (Table 3).

**Table 2:** PK Parameters Following a Single 60‐mg Dose of Atogepant

PK Parameter, Mean (SD)	Severe Hepatic Impairment (n = 8)	Moderate Hepatic Impairment (n = 8)	Mild Hepatic Impairment (n = 8)	Normal Hepatic Function (n = 8)
C_max_ (ng/mL)	543 (197)	529 (227)	666 (372)	589 (248)
T_max_ (h)^a^	1.0 (0.5–3.0)	1.5 (1.0–6.0)	1.8 (1.0–4.0)	1.8 (1.0–3.0)
AUC_0‐t_ (ng·h/mL)	3797 (1397)	3270 (1693)	3473 (1425)	2758 (919)
AUC_0‐∞_ (ng·h/mL)	3836 (1440)	3314 (1684)	3495 (1425)	2780 (924.4)
t_1/2_ (h)	7.5 (3.0)	11.9 (7.6)	8.7 (4.1)	9.4 (3.2)
CL/F (L/h)	17.3 (5.4)	21.3 (8.1)	19.4 (6.6)	24.2 (9.4)
Free fraction in plasma	4.7 (0.9)	3.0 (0.6)	2.7 (0.7)	1.8 (0.5)

AUC_0‐∞_, area under the plasma concentration‐time curve from time 0 to infinity; AUC_0‐t_, area under the plasma concentration‐time curve from time 0 to time t; CL/F, apparent total body clearance of drug from plasma after extravascular administration; C_max_, maximum plasma concentration; PK, pharmacokinetic; SD, standard deviation; T_max_, time to maximum plasma concentration; t_1/2_, apparent terminal elimination half‐life.

a Median (range).

**Table 3:** Atogepant PK Parameters Following a Single 60‐mg Dose of Atogepant in Participants With Severe, Moderate, or Mild Hepatic Impairment Compared With Participants With Normal Hepatic Function

Hepatic Function Category	PK Parameter	Participants With Hepatic Impairment, GM	Participants With Normal Hepatic Function, GM	Impaired/Normal GMR (90%CI)
Severe impairment (n = 8)	C_max_ (ng/mL)	515	539	95.7 (63.9–143.4)
	AUC_0‐t_ (ng·h/mL)	3602	2612	137.9 (99.5–191.1)
	AUC_0‐∞_ (ng·h/mL)	3633	2633	138.0 (99.8–190.7)
Moderate impairment (n = 8)	C_max_ (ng/mL)	475	539	88.2 (58.8–132.1)
	AUC_0‐t_ (ng·h/mL)	2977	2612	114.0 (82.3–157.9)
	AUC_0‐∞_ (ng·h/mL)	3029	2633	115.0 (83.2–159.0)
Mild impairment (n = 8)	C_max_ (ng/mL)	587	539	109.0 (72.7–163.3)
	AUC_0‐t_ (ng·h/mL)	3250	2612	124.4 (89.8–172.4)
	AUC_0‐∞_ (ng·h/mL)	3274	2633	124.3 (89.9–171.8)

AUC_0‐∞_, area under the concentration‐time curve from time 0 to infinity; AUC_0‐t_, area under the plasma concentration‐time curve from time 0 to time t; C_max_, maximum plasma concentration; GM, geometric mean; GMR, geometric mean ratio; PK, pharmacokinetic.

### Atogepant Plasma Protein Binding

The amount of atogepant bound to plasma proteins was 95.3%, 97.1%, 97.4%, and 98.2% in participants with severe, moderate, mild, and no hepatic impairment, respectively. Mean (SD) free fraction of atogepant in plasma across all groups is shown in Table 2.

### Safety

Atogepant appeared to be safe in all hepatic function groups. One AE was reported: an event of mild rhinorrhea occurred after atogepant dosing in a participant with moderate hepatic impairment and was considered by the investigator to be related to atogepant. No SAEs, deaths, or AEs leading to discontinuation were reported. No clinically relevant changes in clinical laboratory parameters, vital signs, or ECG values were observed in any group. There were no Hy's Law cases. One participant met potential Hy's Law criteria; however, this was attributed to underlying AST and total bilirubin elevations already present at screening with minimal change during the study.

## Discussion

A single 60‐mg dose of atogepant was safe in participants with and without hepatic impairment, and no new safety concerns were identified. Compared with participants with normal hepatic function, maximum plasma concentration of atogepant was generally unchanged in participants with severe, moderate, or mild hepatic impairment (–4%, –12%, and +9%, respectively). Overall systemic exposures to atogepant, as measured by AUC_0‐∞_, were slightly higher (14%‐38%) in participants with hepatic impairment compared with those with normal hepatic function, but these differences are not expected to be clinically relevant.^19^ The 60‐mg dose is the highest dose being tested in phase 3 trials and is approximately one‐third of the 170‐mg daily dose tested in a 28‐day hepatic safety study, one‐half of the 60‐mg twice‐daily dose tested in a 12‐week phase 2/3 study, and one‐fifth of the highest single dose of 300 mg atogepant tested in a phase 1 study of the cardiac repolarization effect of supratherapeutic atogepant in healthy participants.^14^, ^19^, ^20^ The safety outcomes in this study, along with those in studies that tested supratherapeutic doses, suggest up to 38% higher systemic exposure of atogepant would not be clinically relevant in people with hepatic impairment.

In healthy adults, atogepant administered orally is rapidly absorbed (median T_max_ of 1 to 2 hours), and systemic exposure increases in a roughly dose‐proportional manner up to a dose of 300 mg. The apparent t_1/2_ of atogepant is approximately 11 hours, and there is no significant accumulation with repeated once‐daily or twice‐daily dosing.

Atogepant was designed to be chemically distinct from telcagepant and MK‐3207 to reduce the potential for reactive metabolites (and thereby reduce DILI) while maintaining potency with lower dosing.^21^ Therapeutic efficacy of atogepant compared with placebo for the prevention of migraine has been demonstrated at daily doses of 10, 30, and 60 mg over 12 weeks in a phase 2/3 clinical trial.^14^, ^15^ Safety results presented from the current trial, together with safety results from the phase 2/3 trial^14^, ^15^ and from a phase 1 trial in healthy adults receiving a 170‐mg daily dose for 28 days,^17^ show continued lack of DILI of atogepant with single or repeat dosing.

Several DDI studies with atogepant have been conducted. Coadministration of atogepant 60 mg and a combination OC (ethinyl estradiol and levonorgestrel [EE/LNG]) was associated with no impact on the PK of EE and about a 19% increase in the AUC of LNG that is not anticipated to be clinically significant.^17^ Another study examining coadministration of sumatriptan 100 mg with atogepant 60 mg found that the PK of sumatriptan was unchanged and atogepant mean C_max_ was reduced by 22% following coadministration, with no change in AUC.^22^ Last, coadministration of atogepant 60 mg with either acetaminophen 1000 mg or naproxen 500 mg demonstrated no statistically significant or clinically relevant change in the PK of atogepant.^23^

Based on the consistent PK profile, overall safety profile, and lack of DILI, these findings support continued testing of atogepant as a treatment for the prevention of migraine. There is a continued unmet need for preventive treatments for people with migraine, and an oral CGRP receptor antagonist may be beneficial for those who prefer oral dosing to infusions or injections of a CGRP monoclonal antibody. Daily oral dosing allows for immediate cessation of therapy in case of AEs in an individual, whereas pharmacological activity of drugs with a long half‐life (eg, monoclonal antibodies) cannot be quickly stopped, and there is no way to rapidly reverse the drug's action.^6^, ^24^ In addition, protein‐based therapeutics can potentially induce an immune response, including infusion reactions,^6^, ^24^ which does not occur with the small‐molecule gepants.

Trial limitations include the sample size, which was relatively small for each hepatic impairment category, although this is in accordance with regulatory guidance and is common with phase 1 trials evaluating the impact of hepatic function on a drug's PK. Although the drug is intended for chronic administration, single‐dose PK was considered adequate because atogepant exhibits linear PK, shows no accumulation with repeated once‐daily dosing, and multiple‐dose PK can be predicted if needed. In addition, PK parameters in the study were calculated based on total (bound and unbound) atogepant concentrations. Increases in free fraction have been shown to lower the therapeutic and toxicity ranges for total plasma concentrations.^25^ Although free fraction (unbound plasma concentration) was more than 2‐fold higher in those with severe hepatic impairment compared with healthy controls, the mean half‐life in this group was lower, suggesting that increased free fraction resulted in relatively rapid elimination of atogepant, thus increasing AUC by only ∼40%. Given the established safety profile of atogepant at a supratherapeutic single dose of 300 mg and multiple doses of 170 mg, it remains unlikely that the changes observed in the severe hepatic impairment subgroup will be clinically relevant. Strengths include the dose of atogepant used in this trial, which is equivalent to the highest clinical dose being tested in phase 3 trials for prevention of migraine. Also, participants representing a spectrum of hepatic impairment severity were evaluated, allowing some generalization to the broader population of people with migraine.

## Conclusion

There was no clinically relevant change in the PK of atogepant in participants with mild, moderate, or severe hepatic impairment compared with participants with normal hepatic function. A single 60‐mg dose of atogepant was safe in participants with hepatic impairment and in participants with normal hepatic function.

## Conflicts of Interest

All authors are or were employees of AbbVie and may hold AbbVie stock.

## Funding

This study was funded by Allergan (prior to its acquisition by AbbVie).

## Data Availability

AbbVie is committed to responsible data sharing regarding the clinical trials we sponsor. This includes access to anonymized, individual, and trial‐level data (analysis data sets), as well as other information (eg, protocols and clinical study reports), as long as the trials are not part of an ongoing or planned regulatory submission. This includes requests for clinical trial data for unlicensed products and indications. These clinical trial data can be requested by any qualified researchers who engage in rigorous, independent scientific research and will be provided following review and approval of a research proposal and statistical analysis plan (SAP) and execution of a data‐sharing agreement (DSA). Data requests can be submitted at any time, and the data will be accessible for 12 months, with possible extensions considered. For more information on the process or to submit a request, visit the following link: https://www.abbvie.com/our‐science/clinical‐trials/clinical‐trials‐data‐and‐information‐sharing/data‐and‐information‐sharing‐with‐qualified‐researchers.html.
